# Clinical Characteristics Associated with Detected Respiratory Microorganism Employing Multiplex Nested PCR in Patients with Presumptive COVID-19 but Negative Molecular Results in Lima, Peru

**DOI:** 10.3390/tropicalmed7110340

**Published:** 2022-10-31

**Authors:** Juan Carlos Gómez de la Torre Pretell, Miguel Hueda-Zavaleta, José Alonso Cáceres-DelAguila, Claudia Barletta-Carrillo, Cesar Copaja-Corzo, Maria del Pilar Suarez Poccorpachi, María Soledad Vega Delgado, Gloria Maria Magdalena Levano Sanchez, Vicente A. Benites-Zapata

**Affiliations:** 1 Roe Clinical Laboratory, Lima 15076, Peru; 2 Facultad de Ciencias de la Salud, Universidad Privada de Tacna, Tacna 23003, Peru; 3 Hospital III Daniel Alcides Carrión-Essalud Tacna, Tacna 23000, Peru; 4 Facultad de Ciencias Biológicas, Universidad Nacional Mayor de San Marcos, Lima 15081, Peru; 5 Red Asistencial Ucayali EsSalud, Pucallpa 25003, Peru; 6 Unidad de Investigación para la Generación y Síntesis de Evidencias en Salud, Universidad San Ignacio de Loyola, Lima 15024, Peru

**Keywords:** respiratory tract infections, common cold, rhinovirus, influenza, COVID-19, polymerase chain reaction, multiplex polymerase chain reaction

## Abstract

The COVID-19 pandemic circumstances have varied the pathogens related to acute respiratory infections (ARI), and most specialists have ignored them due to SARS-CoV-2’s similar symptomatology. We identify respiratory pathogens with multiplex PCR in samples with presumptive SARS-CoV-2 but negative RT-qPCR results. We performed a retrospective transversal study employing clinical data and nasopharyngeal swab samples from patients with suspected clinical SARS-CoV-2 infection and a negative PCR result in a private laboratory in Lima, Peru. The samples were analyzed using the FilmArray™ respiratory panel. Of 342 samples, we detected at least one pathogen in 50% of the samples. The main ones were rhinovirus (54.38%), influenza A(H3N2) (22.80%), and respiratory syncytial virus (RSV) (14.04%). The clinical characteristics were sore throat (70.18%), cough (58.48%), nasal congestion (56.43%), and fever (40.06%). Only 41.46% and 48.78% of patients with influenza met the definition of influenza-like illness (ILI) by the World Health Organization (WHO) (characterized by cough and fever) and the Centers for Disease Control and Prevention (CDC) (characterized by fever and cough and sore throat), respectively. A higher prevalence of influenza was associated with ILI by WHO (aPR: 2.331) and ILI by CDC (aPR: 1.892), which was not observed with other respiratory viruses. The clinical characteristic associated with the increased prevalence of rhinovirus was nasal congestion (aPR: 1.84). For patients with ARI and negative PCR results, the leading respiratory pathogens detected were rhinovirus, influenza, and RSV. Less than half of patients with influenza presented ILI, although its presence was specific to the disease.

## 1. Introduction

Since the announcement by the World Health Organization (WHO), which cataloged the new coronavirus disease (COVID-19) as a public health emergency of international concern (and later a pandemic), an epidemiological variation has occurred in the principal microorganisms that provoke the acute respiratory infections (ARI) globally [1,2,3,4,5]. This phenomenon is probably due to the measures implemented to reduce the transmission and spread of COVID-19, such as using a universal mask, social distancing, and avoiding crowds [6,7]. Consequently, these implementations have correlated with a lower number of incident cases of COVID-19 and indirectly have also decreased the cases of influenza, respiratory syncytial virus (RSV), and rhinovirus [2,3,4,5]. So far, we have not found any studies conducted in Latin American countries with similar criteria. Therefore, the effect of COVID-19 on the etiology of ARI in our region is unknown.

In underdeveloped countries, it is regular to obtain the etiological diagnosis of ARI by employing immunoassays such as rapid antigen tests, direct fluorescent antibodies, or serological antibodies [8]. However, these methods have variable sensitivity and specificity to each microorganism, and human error might be one of the reasons for this fluctuation in these parameters [9].

The molecular techniques based on the polymerase chain reaction (PCR) are fast, sensible, and specific evaluations considered a gold standard for most respiratory viruses. Nonetheless, the main limitations of these methods are that they do not usually detect more than one pathogen and require specialized laboratories for their processing [10]. An alternative for the simultaneous detection of pathogens is the Biofire FilmArray^®^ Respiratory Panel (bioMérieux SA, Marcy-l’Étoile, France), an automated nested multiplex PCR system approved by the Food and Drugs Administration (FDA). This platform detects seventeen viruses and three concurrent bacteria related to respiratory infections, with a sensitivity and specificity above 95% [10,11,12].

The presence of upper respiratory infection symptoms during the COVID-19 pandemic made this disease the primary suspected diagnosis for most general practitioners and specialists [13,14]. Nevertheless, other differential pathogens have not only been ignored as potential causes but also may have changed their incidence, influenced by the new social biosecurity behaviors and other environmental factors. Furthermore, is still unknown if their clinical characteristics associated might have changed as well [15]. To our knowledge, no studies have evaluated these uncertainties in the Latin American region. This study aimed to identify the clinical characteristics associated with respiratory microorganisms identified by multiplex nested PCR in patients with presumptive SARS-CoV-2 infection and negative molecular test.

## 2. Materials and Methods

### 2.1. Study Design and Population

We generated a retrospective transversal study in a private laboratory in Lima, Peru. The period of study was between March and April 2022. We included in the study all the respiratory swab samples from ambulatory patients with IRA symptoms, including cough or sore throat, and also one of the following: fever, headache, rhinorrhea, nasal congestion, diarrhea, general sickness, malaise, or dyspnea. Likewise, we considered a sickness period lower or equal to seven days and a negative SARS-CoV-2 result employing reverse transcription-polymerase chain reaction (RT-PCR) on a nasopharyngeal swab. We excluded all the samples of those patients with indeterminate results or without complete clinical information.

### 2.2. Data Collection and Ethical Aspects

All patients requested informed consent to collect nasopharyngeal swab samples to rule out COVID-19. The clinical-epidemiological investigation sheet for COVID-19 was also obtained from the Ministry of Health of Peru, from which the demographic and clinical data were obtained. The samples were stored in the private laboratory’s biobank as a quality control protocol. The institutional ethics research committee of the Private University of Tacna approved the study’s protocol on 24 April 2022 (Registration Code: 95-FACSA-UI), with the exemption of the informed consent due to its retrospective nature and adherence to the Helsinki standards for research on human subjects. The samples from the laboratory’s biobank were evaluated between 28–29 April 2022.

### 2.3. Processing of Samples and Study Variables

To identify respiratory pathogens, we employed the FilmArray™ Respiratory Panel, an automated nested multiplex PCR system capable of detecting the most common pathogens related to IRA, with a sensibility and specificity of 95% and 99%, respectively [16]. The viruses involved were influenza A (H1N1, H3N2, H1N1-2019 subtypes), influenza B, respiratory syncytial virus, adenovirus, human metapneumovirus, human rhinovirus, enterovirus, coronavirus (229E, HKU1, OC43, NL63) and the human parainfluenza viruses (1, 2, 3, 4). The bacterial pathogens evaluated were Bordetella pertussis, Chlamydophila pneumoniae, and Mycoplasma pneumoniae. The FilmArray ™ platform incorporates the automated sample preparation, nucleic acid extractions, and nested multiplex PCR with automatic detection of the main amplified targets in a system that approximately interprets and presents the results in an hour. The independent variables included in the analysis were age (in years), sex, presence of fever, malaise, cough, sore throat, nasal congestion, and influenza-like illness (ILI) defined by the WHO as a respiratory infection of no more than 10 days characterized by cough and fever and ILI defined by the Center for Disease Control and Prevention (CDC) as fever and cough and/or sore throat [17,18]. The dependent variables were those organisms detected by the FilmArray ™ respiratory panel.

### 2.4. Statistical Analysis

We employed the software STATA v17.0 (StataCorp., College Station, TX, USA) and Prism V 9.2.0 (GraphPad Software, LLC, San Diego, CA, USA) for statistical analysis and representation of results, respectively. We present the qualitative variables as absolute frequency and percentage. The quantitative variables were shown as median and interquartile ranges because the data did not have a normal distribution. According to the circumstances, we compared the mentioned variables with the principal pathogens detected, employing Chi-square, Fisher’s exact test, or Mann–Whitney U test. We considered a value of *p* < 0.05 statistically significant. To evaluate the variables more prevalent with the principal microorganism detected (such as influenza, respiratory syncytial virus, or human rhinovirus), we employed the Poisson regression model with robust variance to estimate the crude prevalence ratio (cPR) and the adjusted prevalence ratio (aPR) with a 95% confidence interval (95% CI).

## 3. Results

We included 342 patient samples, with a median age of 33 years (IQR: 19–41), 5.26% and 12.88% of patients were younger than five years and older than 60 years, respectively. A total of 57.31% of samples came from women and the mean of days with symptoms previous to the etiological diagnostic was two x (IQR:1–3). The principal comorbidities detected on the clinic records according to the samples were heart disease (2.34%), pregnancy (1.46%), and diabetes mellitus type 2 (1.46%). The most common clinical characteristics reported were sore throat (70.18%), cough (58.48%), nasal congestion (56.43%), malaise (43.27%), and fever (40.06%) (Table 1).

**Figure 1:**
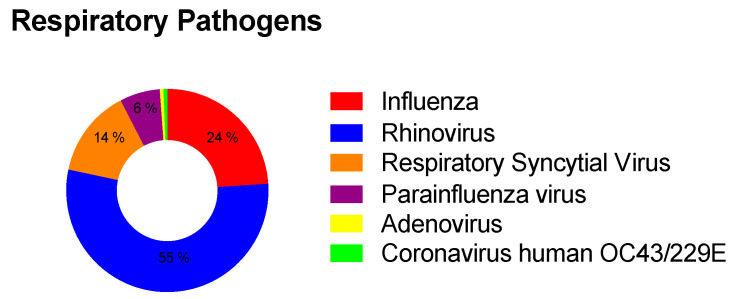
Percentage frequency of pathogens founded with the respiratory panel. This scheme shows the frequency according to the main respiratory microorganisms detected with the respiratory panel that cause acute respiratory infections in patients with negative RT-PCR result for SARS-CoV-2.

A respiratory microorganism was identified in 50% of the patients’ samples. When respiratory microorganisms were detected, the most frequent were rhinovirus (54.38%), influenza A (23.98%), respiratory syncytial virus (14.04%), human parainfluenza virus (6.43%), human coronavirus OC43/229E (1.16%) and adenovirus (0.58%). Notably, we found that 95.12% of influenza were by subtype A(H3N2), and there were coinfections in 2.92% of the patient’s samples with respiratory microorganisms detected (Figure 1).

**Figure 2:**
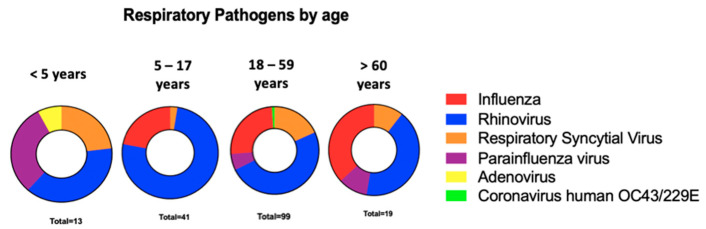
Pathogens founded with the respiratory panel according to age. This scheme shows the frequency according to age group of the main respiratory microorganisms detected with the respiratory panel that cause ARI in patients with negative RT-PCR result for SARS-CoV-2.

We did not find any respiratory microorganisms in 50% of the evaluated samples. In children under five years, rhinovirus and parainfluenza were the most frequent etiologies; meanwhile, in those over 60 years, the most frequent were rhinovirus and influenza (Figure 2).

When we compared the demographic and clinical characteristics according to those principal respiratory microorganisms detected (rhinovirus, influenza A and respiratory syncytial virus), the variables that showed statistical relevance were age, days of symptoms before sampling, and history of heart disease. None of the clinical characteristics showed significant differences (Table 2). We observed that the prevalence of ILI by WHO and CDC in influenza was 41.46% and 48.78%, being more frequent in influenza than in rhinovirus and RSV, although not statistically significant.

In the crude analysis with the Poisson regression model (Table 3), we identified that the history of heart disease (cPR = 3.29; 95% CI: 1.281–8.477; *p* = 0.01) and cough (cPR = 2.20; 95% CI: 1.14–4.34; *p* = 0.02) were more prevalent with influenza. The associated variables with more prevalence of rhinovirus where cough (cPR = 1.93; 95% CI: 1.28–2.89; *p* < 0.001), sore throat (cPR = 1.65; 95% CI: 1.05–2.59; *p* = 0.02) and nasal congestion (cPR = 2.21; 95% CI: 1.46–3.35; *p* < 0.001), while the age was associated with a lower risk (cPR = 0.98; 95% CI: 0.97–0.99; *p* < 0.001). Similarly, nasal congestion was also associated with more prevalence of respiratory syncytial virus (cPR = 2.93; 95% CI: 1.11–7.68; *p* = 0.02). ILI by WHO (cPR = 2.28; 95% CI: 1.29–4.03; *p* = 0.005) and CDC (cPR = 1.85; 95% CI: 1.04–3.28; *p* = 0.03) were associated with a higher prevalence of influenza.

Finally, in the adjusted analysis with the Poisson regression model (Table 4), we identified that ILI by WHO (aPR = 2.33; 95% CI: 1.29–4.18; *p* = 0.005) and ILI by CDC (aPR = 1.89; 95% CI: 1.05–3.40; *p* = 0.03) were associated with presence of influenza. This association was not observed with other respiratory viruses. The characteristics associated with rhinovirus’s presence were nasal congestion (aPR = 1.84; 95% CI: 1.16–2.89; *p* = 0.008) and age (aPR = 0.98; 95% CI: 0.97–0.99; *p* = 0.002) and the only clinical characteristic associated with RSV was nasal congestion (aPR = 2.59; 95% CI: 1.01–6.64; *p* = 0.04).

## 4. Discussion

We found that the most frequent respiratory viruses identified in those patients with ARI and negative SARS-CoV-2 molecular results were rhinovirus (54%), influenza (24%), and respiratory syncytial virus (14%). The clinical characteristics associated with rhinovirus were nasal congestion and a minor age. Only 41.46% and 48.78% of patients with influenza met the definition of ILI by the WHO (characterized by cough and fever) and CDC (characterized by fever and cough and/or sore throat), respectively. However, its presence was associated with a higher prevalence of influenza, which was not observed with other respiratory viruses.

Patients with COVID-19 may present with symptoms identical to ILI. A significant decrease in the prevalence of other respiratory viruses has been evidenced by the emergence of SARS-CoV-2 in multiple ARI surveillance centers. Furthermore, an essential variation in the age of these cases was observed, being mainly in adults and the elderly more than in children [1,2,3,4,5,19], which may be secondary to the viral interference caused by SARS-CoV-2 and to the measures implemented to reduce the transmission and spread of COVID-19 [6,7]. The positivity rate found in our study was 50%, which was higher than other studies [10,20,21,22], even though we used the same nested multiplex PCR platform for the diagnosis. Nevertheless, the median time of sickness until the moment of the molecular diagnostic was two days. This short period might relate to a higher viral load and heightened test sensitivity during the evaluation. As well as in other studies, the positivity rate was bigger in patients under five years old (66.67%) and diminished progressively with age [23,24,25]. Probably because those under five years present a higher incidence of ARI (on average, six episodes per year) [26] added to a greater spread due to fomites, close contact with an infected patient, and an immature immune system.

The main microorganism causing ARI in all the age groups of the cohort study was rhinovirus, a common virus in all seasons [24]. It spreads due to fomites, and even when its infectivity on surfaces decreases in a matter of hours [25], being a non-enveloped virus gives it better resistance to alcohol-based disinfectants, widely used as a protective measure against COVID-19. The second most frequent microorganism detected was the influenza virus, in which subtype A(H3N2) was the predominant agent (mainly in adults). Its presence coincided with the epidemiological surveillance data of Peru that reported it during week 49 of 2021 [27].

Only 3% of the samples showed coinfections, a number that was minor compared to previous studies [10,28,29,30], and the most frequent mixture was that between rhinovirus/influenza. We did not detect any of the bacteria included in the FilmArray™ Respiratory Panel in the study’s samples. This particularity might reassert the FilmArray’s utility as a device for antimicrobial stewardship, reducing unnecessary antibiotics prescriptions [31] and giving results faster than other methods [32]. The Infectious Diseases Society of America (IDSA) recommends using rapid diagnostic tests for respiratory viruses as an antimicrobial stewardship strategy to reduce the inappropriate use of antibiotics [33]. Although there are discrepancies in this regard, some authors have observed that multiplex molecular assays lessen the use and time of empirical antibiotic therapy, whether in patients treated as outpatients [34], hospitalized [35], or in the emergency room [36].

Bacterial co-infections occur in only 0.5% of influenza infections but can complicate up to 34% and 43% of patients with influenza [37] and RSV in intensive care [38], respectively. In contrast, COVID-19 has shown a prevalence of co-infections between 7 to 14% [39], reaching up to 54% in patients admitted to intensive care [40,41,42].

Even when the WHO routinely suggests employing the association of fever and cough as a syndromic reference of influenza, we found that only 41.46% of patients met these criteria. In our study, among patients with influenza infection, 51% presented fever and 75% cough. This last symptom was the only manifestation associated with an influenza infection in crude analysis. This symptom was also related to other viruses, such as the human rhinovirus. These findings differ from the study results from Nateghian et al., where the fever’s frequency was 82%, and the cough was 43% for the patients with influenza [43]. However, they reported a discreet increased frequency of fever in the A(H1N1) subtype compared to the A(H3N2), which was the principal subtype in our study.

Chow et al. established that the sensitivity for the influenza-like illness clinical criteria according to WHO was only 38.5%, making us question its actual utility [44]. Nonetheless, although ILI was present in less than half of the patients with influenza in our study, its presence was the only statistically significant variable associated with influenza in the adjusted analysis. This association was not observed with other respiratory viruses, which would reflect its high specificity and could address the need for empiric antiviral therapy such as oseltamivir in its presence, mainly with the definition proposed by the WHO in settings of limited resources.

We observed that the presence of nasal congestion was the only clinical variable significantly associated with the presence of rhinovirus. It has been reported that patients infected with rhinovirus present nasal hyperreactivity to histamine and methacholine, characterized by a secretory response and increased sneezes [45,46]. Likewise, the symptoms seem to correlate with the viral load of rhinovirus in the nasopharynx [47], being higher in the first days of illness. Our findings could suggest that in the presence of nasal congestion and absence of ILI, the main microorganisms could be rhinovirus, mainly in younger patients.

This study has some limitations. Firstly, the study’s retrospective nature made it impossible to perform a clinical follow-up with the patient after its molecular results and even know its outcome (hospitalization, survival, or death). Secondly, the study performed the molecular evaluations in a unique institution, so we cannot extrapolate the results to other populations. Thirdly, the study period was short, which did not allow us to evaluate the prevalence of the respiratory microorganisms according to the year’s seasons. Finally, we analyzed samples of those patients who looked for assistance in our institution, which might generate a selection bias; however, we minimized this effect because we evaluated all the samples received.

## Figures and Tables

**Table 1:** Main variables. This table shows the demographic and clinical characteristics of the population studied.

Variable	(n = 342)
Age (years) ^1^	33.39 (19.70–47.17)
≤5 years (%)	18 (5.26)
6–17 years (%)	61 (17.84)
18–59 years (%)	221 (64.62)
≥60 years (%)	42 (12.28)
Positivity by age categorized	
≤5 years (%)	12 (66.67)
6–17 years (%)	41 (67.21)
18–59 years (%)	99 (44.79)
≥60 years (%)	19 (45.23)
Sex	
Female (%)	196 (57.31)
Male (%)	146 (42.69)
Days of symptoms ^1^	2 (1–3)
Comorbidities	
Pregnancy (%)	5 (1.46)
Cardiopathy (%)	8 (2.34)
Diabetes (%)	5 (1.46)
Chronic kidney disease (%)	2 (0.58)
Chronic pulmonary disease (%)	3 (0.88)
Cancer (%)	1 (0.29)
Clinical characteristics	
Fever (%)	137 (40.06)
General discomfort (%)	148 (43.27)
Cough (%)	200 (58.48)
Sore throat (%)	240 (70.18)
Respiratory distress (%)	45 (13.16)
Nasal congestion (%)	193 (56.43)
Diarrhea (%)	34 (9.94)
Nausea (%)	23 (6.73)
Headache (%)	62 (18.13)
Irritation (%)	5 (1.46)
Myalgias (%)	41 (11.99)
Abdominal pain (%)	11 (3.22)
Thorax pain (%)	15 (4.39)
Arthralgias (%)	8 (2.34)
Influenza-like Illness by WHO (%)	81 (23.68)
Influenza-like Illness by CDC (%)	116 (33.92)
Pathogens detected (%)	171 (50.00)
Rhinovirus (%)	93 (54.38)
Influenza A/B (%)	41 (23.98)
A(H3N2)	39 (95.12)
Respiratory Syncytial Virus (%)	24 (14.04)
Parainfluenza (%)	11 (6.42)
Adenovirus (%)	1 (0.58)
Human Coronavirus OC43	1 (0.58)
Human Coronavirus 229E	1 (0.58)
Co-infections (Influenza/Rhinovirus)	5 (2.92)

^1^ Median and interquartile range.

**Table 2:** Variables in relation to principal pathogens. This table compares the demographic and clinical characteristics of the study population with the main respiratory viruses detected.

Variable	Rhinovirus (n = 89)	Influenza (n = 41)	RSV (n = 22)	*p*-Value
Demographic characteristics				
Age (years) *	25.67 (9.69–35.28)	32.87 (21.24–44.19)	35.41 (23.69–45.27)	0.028 ^a^
<5 years (%)	5 (5.61)	0 (0.0)	3 (13.63)	0.022 ^b^
6–17 years (%)	30 (33.70)	9 (21.95)	1 (4.55)	
18–59 years (%)	47 (52.80)	25 (60.98)	16 (72.72)	
>60 years (%)	7 (7.89)	7 (17.07)	2 (9.10)	
Sex				0.272 ^b^
Female	54 (60.68)	20 (48.78)	10 (45.45)	
Male	35 (39.32)	21 (51.22)	12 (54.55)	
Days of symptoms before sampling *	2 (1–3)	3 (2–4)	2.5 (2–4)	0.028 ^a^
Comorbidities				
Pregnancy (%)	0 (0.0)	1 (2.43)	0 (0.0)	0.999 ^c^
Cardiopathy (%)	0 (0.0)	3 (7.31)	0 (0.0)	0.038 ^c^
Diabetes (%)	0 (0.0)	0 (0.0)	1 (4.54)	0.145 ^c^
Chronic kidney disease (%)	1 (1.12)	0 (0.0)	0 (0.0)	0.999 ^c^
Chronic pulmonary disease (%)	1 (1.12)	0 (0.0)	0 (0.0)	0.999 ^c^
Clinical characteristics				
Fever (%)	32 (35.95)	21 (51.21)	7 (31.81)	0.186 ^b^
General malaise (%)	43 (48.31)	17 (41.46)	9 (40.90)	0.691 ^b^
Cough (%)	65 (73.03)	31 (75.60)	16 (72.72)	0.947 ^b^
Sore throat (%)	70 (78.65)	32 (78.04)	19 (86.36)	0.694 ^b^
Respiratory distress (%)	7 (7.86)	8 (19.51)	5 (22.27)	0.067 ^c^
Nasal congestion (%)	67 (75.28)	24 (58.53)	17 (77.27)	0.129 ^b^
Diarrhea (%)	9 (10.11)	5 (12.19)	3 (13.63)	0.816 ^c^
Nausea (%)	4 (4.49)	3 (7.31)	2 (9.10)	0.568 ^c^
Headache (%)	16 (17.97)	5 (12.19)	6 (27.27)	0.307 ^c^
Irritation (%)	1 (1.12)	2 (4.87)	1 (4.55)	0.236 ^c^
Myalgias (%)	8 (8.98)	6 (14.63)	4 (18.18)	0.324 ^c^
Abdominal pain (%)	1 (1.12)	0 (0.0)	0 (0.0)	0.999 ^c^
Thorax pain (%)	2 (2.24)	2 (4.87)	11 (50.00)	0.533 ^c^
Arthralgias (%)	0 (0.0)	1 (0.50)	1 (0.50)	0.170 ^c^
Influenza-like illness by WHO	24 (26.97)	17 (41.46)	6 (27.27)	0.232 ^b^
Influenza-like illness by CDC	31 (34.82)	20 (48.78)	7 (31.82)	0.253 ^b^

* = Median and interquartile range, ^a^ = U-Mann Whitney test, ^b^ = chi-squared test, ^c^ = Fisher’s exact test, RSV = Respiratory Syncytial Virus, WHO = World Health Organization; CDC = Center for Disease Control and Prevention.

**Table 3:** Main clinical characteristics in relation to the pathogens detected. This table shows the Poisson regression analysis to evaluate crude prevalence ratio of respiratory virus.

Variable	Influenza		Rhinovirus		Respiratory Syncytial Virus	
	cPR (95% CI)	*p*-Value	cPR (95% CI)	*p*-Value	cPR (95% CI)	*p*-Value
Age	1.006 (0.991–1.020)	0.409	0.980 (0.970–0.990)	<0.001	1.000 (0.983–1.017)	0.994
Cardiopathy	3.296 (1.281–8.477)	0.013	0.971 (0.272–3.091)	0.890	-	-
Fever	1.571 (0.885–2.788)	0.123	0.862 (0.599–1.239)	0.424	0.616 (0.262–1.448)	0.267
General discomfort	0.928 (0.517–1.665)	0.803	1.177 (0.832–1.665)	0.357	1.109 (0.510–2.407)	0.793
Cough	2.201 (1.114–4.346)	0.023	1.931 (1.287–2.896)	<0.001	1.420 (0.624–3.231)	0.403
Sore throat	1.511 (0.747–3.053)	0.250	1.655 (1.057–2.591)	0.028	2.975 (0.905–9.769)	0.072
Nasal congestion	1.089 (0.607–1.954)	0.773	2.219 (1.468–3.354)	<0.001	2.933 (1.119–7.685)	0.029
Influenza-like illness by WHO	2.282 (1.290–4.035)	0.005	1.184 (0.805–1.741)	0.389	1.074 (0.440–2.618)	0.875
Influenza-like illness by CDC	1.855 (1.048–3.283)	0.034	1.071 (0.746–1.538)	0.708	0.802 (0.342–1.881)	0.612

cRR: crude Prevalence Ratio; 95% CI: 95 percent confidence interval; WHO: World Health Organization; CDC: Center for Disease Control and Prevention.

**Table 4:** Main clinical characteristics in relation to the pathogens detected. This table shows the Poisson regression analysis to evaluate adjusted prevalence ratio of respiratory virus.

Variables	Influenza		Rhinovirus		Respiratory Syncytial Virus	
	aPR (95% CI)	*p*-Value	aPR (95% CI)	*p*-Value	aPR (95% CI)	*p*-Value
Age	1.007 (0.992–1.023)	0.335	0.983 (0.973–0.993)	0.002	1.005 (0.987–1.024)	0.545
Cardiopathy	3.007 (0.992–1.023)	0.074	-	-	-	-
General malaise	0.028 (0.425–1.511)	0.496	1.042 (0.742–1.462)	0.812	0.833 (0.391–1.774)	0.669
Sore throat	1.684 (0.829–3.423)	0.149	1.256 (0.791–1.993)	0.333	2.376 (0.715–7.893)	0.157
Nasal congestion	0.979 (0.515–1.858)	0.949	1.840 (1.169–2.897)	0.008	2.591 (1.010–6.645)	0.048
Influenza-like illness by WHO	2.331 (1.298–4.183)	0.005	0.917 (0.628–1.339)	0.655	0.911 (0.382–2.170)	0.835
Influenza-like illness by CDC	1.892 (1.051–3.409)	0.034	0.858 (0.604–1.219)	0.394	0.717 (0.304–1.691)	0.448

cRR: crude Prevalence Ratio; 95% CI: 95 percent confidence interval; WHO: World Health Organization; CDC: Center for Disease Control and Prevention.

## Data Availability

The data analyzed in this manuscript, as well as its definitions, can be downloaded at the following: https://doi.org/10.17632/54nj8895px.1.
